# Facial pemphigus vegetans mimicking squamous cell carcinoma: when dermoscopy confuses the diagnosis^☆^

**DOI:** 10.1016/j.abd.2024.05.009

**Published:** 2025-01-22

**Authors:** Nelson Lobos, Francisca Reculé, Macarena Stevenson, Valentina Darlic, Dan Hartmann, Alex Castro

**Affiliations:** a Dermato-Oncology Department, Instituto Nacional del Cáncer, Santiago, Chile; b Dermatology Department, Facultad de Medicina, Universidad de Chile, Santiago, Chile; c Dermatology Department, Clínica Alemana, Universidad del Desarrollo, Santiago, Chile; d Faculty of Medicine, Universidad del Desarrollo, Santiago, Chile; e Faculty of Medicine, Universidad Finis Terrae, Santiago, Chile; f Pathology Department, Clínica Alemana, Universidad del Desarrollo, Santiago, Chile

Dear Editor,

Pemphigus vegetans (PVeg) is a rare variant of pemphigus vulgaris (PV) (1%–2% of cases) characterized by flaccid bullae or pustules that erode to form hypertrophic plaques and vegetating masses.^1^ Generally, the lesions are multifocal and localized on flexures, periorificial areas, and oral mucosa.^1^, ^2^ It has two clinical forms that have been described in the literature: the Neumann type and the Hallopeau type.^2^ The diagnosis is made based on clinical features, but the biopsy is mandatory to confirm it. There are no dermatoscopic reports of PVeg, but the presence of pustules, micro-vesicles, and erosions can guide us in its initial stage. Histological examination shows acantholysis, epidermal hyperplasia, papillomatosis, and intraepidermal eosinophilic and neutrophilic abscesses. Direct Immunofluorescence (DIF) demonstrates intercellular deposition of IgG and C3.^1^, ^3^

Herein, we report a challenging case presenting as a solitary facial hyperkeratotic plaque of PVeg without oral mucosal involvement initially misdiagnosed as a squamous cell carcinoma (SCC) by clinical, dermoscopy, and histological examination.

We present, a sixty-three-year-old male who was referred to our dermatology department for an asymptomatic recurrent lesion of the right frontal area which had been present for two years. Physical examination showed a 2 × 2.5 cm well-demarcated hyperkeratotic solitary plaque with an eroded surface (Fig. 1A). No intertriginous or oral mucosa involvement was seen. Dermoscopy revealed a predominantly white background with surface scale, multiple different-sized keratin-filled follicular ostia, and white perifollicular circles surrounded by erythema (Fig. 1B). He had an incisional 4 mm punch biopsy, performed in an external health center, that showed an invasive SCC. The lesion was completely excised with Mohs micrographic surgery. The final pathology report discarded SCC and suggested warty dyskeratoma. Six months later, the lesion recurred as a superficial scaly plaque over the previous scar (Fig. 2A). Dermoscopy showed mainly follicular plugs, irregular pigmentation of follicular openings, and pseudo network areas (Fig. 2B). The original slides were reviewed in our pathology department. Microscopic examination revealed epidermal hyperplasia, papillomatosis, acantholysis, and intraepidermal eosinophilic and neutrophilic abscesses (Fig. 3A‒C). An additional biopsy for DIF demonstrated intercellular deposition of C3 and IgG (Fig. 3D). Histopathological findings were compatible with pemphigus vegetans. Confirming the diagnostic of PVeg Neumann type.

**Fig. 1:**
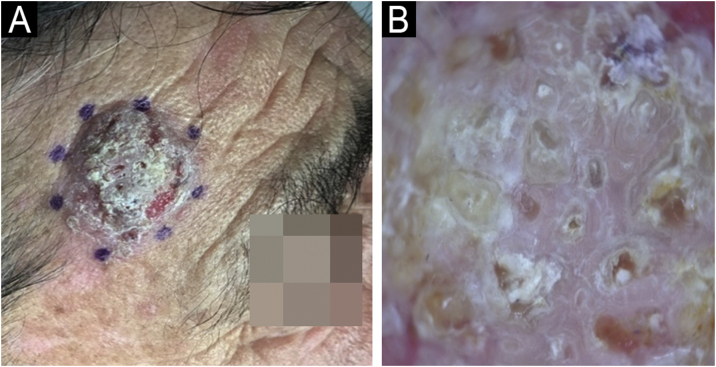
(A) Well-demarcated hyperkeratotic plaque with an eroded surface. (B) Dermoscopy reveals a white background with surface scale, multiple keratin-filled follicular ostia, white perifollicular circles surrounded by erythema, and white structureless areas. Some red areas attributable to bleeding and/or dense vascularity are seen at the periphery.

**Fig. 2:**
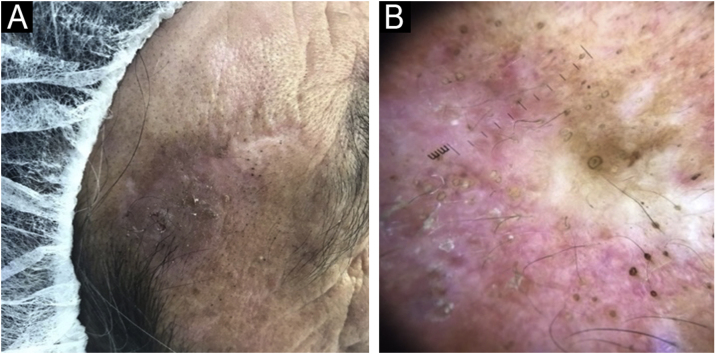
(A) Recurrence. Scaly plaque over the previous scar. (B) Dermoscopy shows a milky-red background with pseudonetwork between target-like keratin-filled follicular ostia. An irregular pigmentation of follicular openings and some white structureless areas are also seen.

**Fig. 3:**
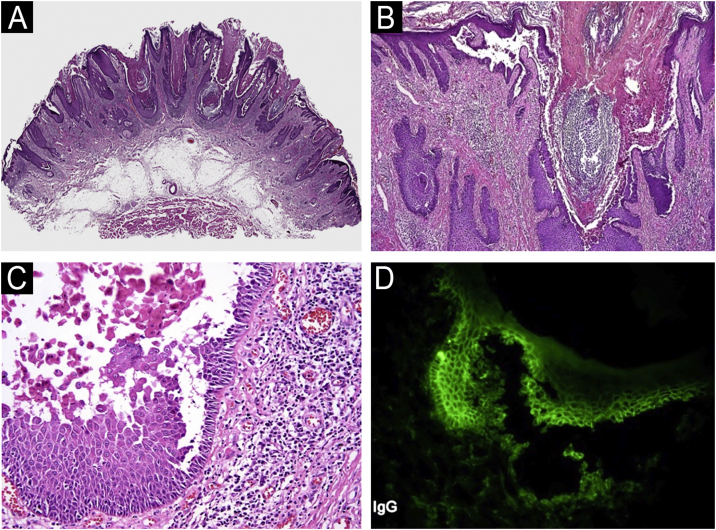
(A‒C) Light microscopy of the first biopsy reveals epidermal hyperplasia, papillomatosis, and acantholysis. (D) Direct Immunofluorescence demonstrates intercellular deposition of IgG.

PVeg corresponds to an autoimmune disease characterized by flaccid bullae or pustules that erode to form hypertrophic plaques involving predominantly skin flexures and mucous membranes.^4^

Two clinical forms have been described in the literature: Hallopeau type and Neumann type. The Hallopeau type is a milder form characterized by pustules and may cure as vegetating plaques or have spontaneous remission.^2^ The pustules are the primary lesions, followed by vegetations, with a lack of bullae, going through a benign course and few relapses.^5^ The Neumann type has a worse prognosis, and it’s characterized by vesicular and erosive lesions that evolve into vegetative plaques, a more severe clinical course, and less response to treatment.^2^ Also, in the Neumann type, the denuded area tends to heal with papillomatous formations and is characterized to begin and end with bullae.^6^ This is compatible with the history and examination of our patient.

Clinically, the intertriginous area is the most frequently involved site for PVeg as well as mucous membranes, and the occurrence of PVeg in non-intertriginous areas is extremely rare.^6^ To our knowledge, there are only a few reported cases of PVeg with exclusive facial localization^6^, ^7^ and even fewer mimicking malignancies.^8^

The differential diagnosis includes the vegetating lesions such as bullous pemphigoid or immunoglobulin A (IgA) pemphigus, the chronic inflammatory plaques of Hailey-Hailey disease, and especially vegetating pyoderma.^1^ Frequently, patients with non-intertriginous PVeg are initially misdiagnosed and improperly treated for months or even years;^5^ this has happened in our case where facial lesions become a diagnostic challenge.

The histopathology and immunofluorescence studies together play a key role in diagnosing the disease.^5^ The treatment of this disease is with systemic steroids in the form of oral prednisolone or injectable dexamethasone, with excellent response to steroids.^1^, ^2^, ^5^

In conclusion, PVeg is a rare variant of PV that occasionally presents as a solitary plaque, which can be confused with a tumor on clinical and dermatoscopic examination. Furthermore, a partial biopsy of the lesion may be misinterpreted as squamous cell carcinoma by an inexperienced pathologist. PVeg should be considered in the differential diagnosis if recurrence develops after complete removal. Evaluation by an experienced pathologist and DIF allows for correct diagnosis and proper management.

## Financial support

None declared.

## Authors’ contributions

Nelson Lobos: Approval of the final version of the manuscript; critical literature review; manuscript critical review; preparation and writing of the manuscript.

Francisca Reculé: Critical literature review; manuscript critical review; preparation and writing of the manuscript.

Macarena Stevenson: Critical literature review; manuscript critical review; preparation and writing of the manuscript.

Valentina Darlic: Critical literature review; manuscript critical review; preparation and writing of the manuscript.

Dan Hartmann: Critical literature review; manuscript critical review; preparation and writing of the manuscript.

Alex Castro: Critical literature review; manuscript critical review; preparation and writing of the manuscript.

## Conflicts of interest

None declared.
